# Exosomal Non-Coding RNAs: Novel Regulators of Macrophage-Linked Intercellular Communication in Lung Cancer and Inflammatory Lung Diseases

**DOI:** 10.3390/biom13030536

**Published:** 2023-03-15

**Authors:** Xingning Lai, Jie Zhong, Boyi Zhang, Tao Zhu, Ren Liao

**Affiliations:** 1 Department of Anesthesiology, West China Hospital, Sichuan University, Chengdu 610041, China; 2 Research Unit for Perioperative Stress Assessment and Clinical Decision, Chinese Academy of Medical Sciences (2018RU012), West China Hospital, Sichuan University, Chengdu 610041, China

**Keywords:** exosomal ncRNAs, macrophage, lung cancer, inflammatory lung diseases, intercellular communication

## Abstract

Macrophages are innate immune cells and often classified as M1 macrophages (pro-inflammatory states) and M2 macrophages (anti-inflammatory states). Exosomes are cell-derived nanovesicles that range in diameter from 30 to 150 nm. Non-coding RNAs (ncRNAs), including microRNAs (miRNAs), long noncoding RNAs (lncRNAs), and circular RNAs (circRNAs), are abundant in exosomes and exosomal ncRNAs influence immune responses. Exosomal ncRNAs control macrophage-linked intercellular communication via their targets or signaling pathways, which can play positive or negative roles in lung cancer and inflammatory lung disorders, including acute lung injury (ALI), asthma, and pulmonary fibrosis. In lung cancer, exosomal ncRNAs mediated intercellular communication between lung tumor cells and tumor-associated macrophages (TAMs), coordinating cancer proliferation, migration, invasion, metastasis, immune evasion, and therapy resistance. In inflammatory lung illnesses, exosomal ncRNAs mediate macrophage activation and inflammation to promote or inhibit lung damage. Furthermore, we also discussed the possible applications of exosomal ncRNA-based therapies for lung disorders.

## 1. Introduction

Resident tissue macrophages remain one of the most frequent immune cells and develop from embryonic progenitors or bone marrow monocytes [1]. The functions of resident tissue macrophages include the removal of dead cells and other cell debris, tissue immune surveillance, and maintenance of tissue homeostasis [2]. Alveolar macrophages (AMs), interstitial macrophages (IMs), and recruited macrophages are macrophages found in lung tissues. AMs shield the alveoli and airways from inhaled pathogens and particles. On the vascular and lung interstitium, IMs have immune surveillance effects [3,4]. Recruited macrophages are derived from recruited monocytes during lung inflammation [4]. Alveolar epithelial cells (AECs) serve as lung tissue barriers to protect from external and internal challenges. AMs can interact with AECs to maintain lung homeostasis [5]. Macrophages are activated in response to tissue damage and go through considerable phenotypic and functional changes to engage in tissue repair and regeneration. Additionally, dysregulated macrophage populations can impede tissue regeneration and cause pathological fibrosis [6]. Macrophages are related to various inflammatory respiratory illnesses, including acute lung injury (ALI) [7], asthma [8], and pulmonary fibrosis [9]. Currently, cancer ranks as the primary cause of disease-related deaths throughout the world [10]. The tumor microenvironment (TME), which drives the development of cancer, is complex and contains stromal cells, fibroblasts, endothelial cells, and immune cells [11]. Tumor-associated macrophages (TAMs) are macrophages that infiltrate the TME after being recruited by tumor cells. TAMs originate from resident macrophages and circulating monocytes [12,13]. TAMs are known to support cancer growth, migration, invasion, angiogenesis, metastasis, and resistance to chemotherapy and radiotherapy [14,15]. TAM presence is also strongly linked to a poor prognosis in cancer patients [16].

Extracellular vesicles (EVs) are membranous particles released by cells into the extracellular space. Exosomes (30 to 150 nm in diameter), shed microvesicles (MVs) (150 to 1000 nm in diameter), and apoptotic bodies (50 to 5000 nm in diameter) are the three subtypes of EVs that are separated based on size range and biogenesis [17]. Exosomes are generated from the endocytic pathway involved in plasma membrane budding and the formation of multivesicular endosomes containing intraluminal vesicles [18]. Exosomes govern cell-cell communication by transporting bioactive cargoes (e.g., proteins, lipids, and nucleic acids) from donor cells to recipient cells [19]. Exosomes contain a lipid bilayer membrane that protects against enzymatic degradation of these intracellular contents [20]. Non-coding RNAs (ncRNAs) are RNA transcripts without the potential to encode proteins. ncRNAs contain microRNAs (miRNAs), long noncoding RNAs (lncRNAs), and circular RNAs (circRNAs). miRNAs are small single-stranded RNAs with a length of 17~25 nucleotides. miRNAs recognize the 3′untranslated region (3′UTR) of messenger RNA (mRNA) and suppress gene expression by repressing mRNA translation or degrading mRNA [21,22]. Each miRNA can target various mRNAs, thereby mediating the expression of several genes. Each gene can also be regulated by multiple miRNAs [23]. LncRNAs are large RNA transcripts with a length of more than 200 nucleotides. LncRNAs include the following different subtypes: sense lncRNA, antisense lncRNA, bidirectional lncRNA, intergenic lncRNA, and intronic lncRNA [24]. LncRNAs share miRNA response elements and function as competitive endogenous RNAs (ceRNAs) to reduce miRNA activity, ultimately disrupting the connection between miRNAs and their target genes [25]. circRNAs are produced via back-splicing and have closed-loop structures without 5′ end caps and 3′ end poly(A) tails. circRNAs originate from exons or introns. circRNAs can also act as ceRNAs to sponge miRNAs and inhibit miRNA repression on its targeted mRNAs [26,27]. ncRNAs can be loaded into exosomes. There is substantial evidence that exosomal ncRNAs are critical for human health. Exosomal ncRNA abnormalities, however, have been related to many human diseases, such as cancers, autoimmune disorders, infectious diseases, etc. [28]. Exosomal ncRNAs produced by TAMs, for instance, potentiate tumor proliferation, invasion, migration, angiogenesis, chemoresistance, and immunosuppression [29]. The application of circulating exosomal ncRNAs could be a promising noninvasive approach for cancer prognosis prediction and early cancer diagnosis [30,31].

In this review, we discussed the functional roles of the exosomal ncRNAs in macrophage-linked intercellular communication in lung cancer and inflammatory lung diseases, including ALI, asthma, and pulmonary fibrosis. In lung TME, exosomal ncRNAs govern bilateral communication between TAMs and lung tumor cells. In inflammatory lung diseases, exosomal ncRNAs mediate the crosstalk between macrophages and lung tissue cells. Furthermore, exosomal ncRNAs have the promising potential to function as diagnostic and prognostic biomarkers in lung diseases.

## 2. Overview of Macrophage Polarization Phenotypes

According to the M1/M2 paradigm, macrophages can be activated into two distinct populations: M1 macrophages (classically activated state) and M2 macrophages (alternatively activated state) [32]. M1 macrophages are induced by interferon-γ (IFN-γ), tumor necrosis factor-α (TNF-α), and lipopolysaccharide (LPS). M2 macrophages are classified into three subtypes: M2a, M2b, and M2c. M2a macrophages are triggered by interleukin-4 (IL-4) and IL-13. M2b macrophages are driven by immune complexes, toll-like receptor (TLR) ligands, and IL-1 receptor (IL-1R) ligands. M2c macrophages are stimulated by IL-10, transforming growth factor-β (TGF-β), and glucocorticoids [33]. M1 macrophage polarization is characterized by the expression of phenotypic markers and molecules linked to antigen presentation, such as major histocompatibility complex class II (MHC II), CD16, CD32, CD80, CD86, and IL-1R. M1 macrophages also release many pro-inflammatory molecules, including IL-1, IL-6, IL-12, IL-23, inducible nitric oxide synthase (iNOS), and matrix metalloproteinase 12 (MMP12) [34]. M2 macrophages have high levels of CD163, CD200R, CD204, and CD206 expression [35,36]. M2 macrophages also produce anti-inflammatory cytokines, such as IL-10, arginase-1 (Arg1), C-C motif chemokine ligand 24 (CCL24), CCL22, and others [35]. M1 macrophages are related to T helper 1 (Th1) responses, type I inflammation, bacterial killing, and tumor resistance [33]. M2a macrophages induce Th2 responses, allergy, anti-inflammatory response, and tissue remodeling [34]. M2b macrophages involve Th2 activation, tumor progression, and immunomodulation [37]. M2c macrophages lead to immunosuppression, matrix deposition, and tissue remodeling [37,38]. In summary, to accurately describe the phenotypic characterization of macrophages, it is essential to consider all described markers comprehensively. Individual markers are limited to distinguishing macrophage phenotypes, considering that distinct cellular subsets can share some molecules [39].

## 3. Exosomal ncRNAs Regulate Macrophage-Linked Intercellular Communication in Lung Cancer

Lung cancer is the most frequent cancer based on current diagnoses and the leading cause of cancer-related mortality globally [40]. Lung cancer can be divided into small cell lung cancer (SCLC) (15%) and non-small cell lung cancer (NSCLC) (85%). NSCLC contains lung adenocarcinoma (40%), lung squamous cell carcinoma (30%), and large cell carcinoma (15%) [41]. Lung cancer risk factors include genetic mutations and environmental interactions, such as cigarette smoking, radiation exposure, and toxic substances [42]. Presently, metastasis and recurrence are the main causes of death for lung cancer patients. Approximately two-thirds of patients have metastatic lesions when they are first diagnosed with lung cancer [43]. Although there are many lung cancer therapies (surgical resection, chemotherapy, radiotherapy, etc.), the overall five-year survival rate of lung cancer patients is still as low as 17.7% [44], and the overall five-year survival rate of patients with distant organ metastasis is 4.5% [45]. TAMs, together with cancer stem cells, cancer cells, fibroblasts, T cells, B cells, and natural killer (NK) cells, form an immune-suppressive TME. TAMs are related to cancer-related inflammation and drive cancer therapy tolerance, cancer recurrence, and cancer distant metastasis [46,47]. M1 TAMs inhibit lung cancer development. M2 TAMs reprogram lung TME and promote immune evasion of lung cancer cells [48]. Pritchard et al. discover that lung tumor cell-derived exosomes boost macrophage polarization into the M2 state [49]. Zhu et al. reveal that macrophage-derived apoptotic bodies strengthen lung tumor cell proliferation [50]. Additionally, exosomes derived from M2 macrophages boost drug resistance in NSCLC [51]. In summary, lung tumor cell-TAM crosstalk is a major driver of lung tumor progression. Exosomes function as essential intercellular messengers to regulate cell-to-cell communication between lung tumor cells and TAMs.

### 3.1. The Impacts of Exosomal miRNAs on Macrophage-Linked Intercellular Communication

#### 3.1.1. Exosomal miRNAs from Macrophages Can Be Transferred into Lung Tumor Cells

Compared to M1 macrophages, miR-942 expression is greater in M2 macrophages. In lung adenocarcinoma tissues, increased miR-942 expression is correlated with strong TAM infiltration. miR-942 over-expression eliminates forkhead box subfamily O 1 (FOXO1) expression to boost β-catenin expression [52]. miR-942 in M2 macrophages can be transported to SPC-A1 cells and H1299 cells via exosomes. Subsequently, through the FOXO1-β-catenin axis, exosomal miR-942 stimulates lung tumor cell migration and cell invasion and facilitates angiogenesis [52]. Additionally, miR-942 from M2 macrophage exosomes also accelerates lung tumor metastasis and promotes angiogenesis in vivo. This study also indicates that in individuals with lung adenocarcinoma, high miR-942 expression is associated with a poor prognosis [52]. miR-501-3p is more abundant in M2 macrophage exosomes than in THP-1 cell (human mononuclear cell line) exosomes. Exosomes from M2 macrophages with miR-501-3p can be transported to A549 and SPC-A1 cells. By decreasing WD repeat domain 82 (WDR82), exosomal miR-501-3p suppresses lung tumor cell apoptosis and promotes lung tumor cell proliferation, cell migration, and cell invasion [53]. Epithelial-mesenchymal transition (EMT), a process through which epithelial cells lose cell-cell connections and turn into mesenchymal cells, is associated with wound healing, pulmonary fibrosis, and cancer metastasis [54]. In the exosomes produced by M2 macrophages, miR-155 and miR-196a-5p are substantially expressed. miR-155 and miR-196a-5p bind to the 3′UTR of Ras association domain family member 4 (RASSF4) [55]. M2 macrophage-delivered exosomal miR-155 and miR-196a-5p reduce RASSF4 to facilitate cell migration, invasion, and EMT in A549 cells. Furthermore, exosomal miR-155 and miR-196a-5p also potentiate NSCLC metastasis in vivo [55]. The E3 ubiquitin ligase neural precursor cell-expressed developmentally down-regulated gene 4-like (NEDD4L) enhances c-Myc ubiquitination and reduces c-Myc protein levels. Compared to M0 macrophages, miR-3679-5p is over-expressed in M2 macrophages. M2 macrophage exosomal miR-3679-5p increases c-Myc protein expression via inhibiting NEDD4L, which can promote aerobic glycolysis and thereby enhance chemoresistance in A549 cells [56]. AKT (also known as protein kinase B) is a serine/threonine kinase and consists of three different isoforms: AKT1, AKT2, and AKT3 [57]. miR-3917 is enriched in lung cancer tissues. Exosomal miR-3917 from M2 macrophages accelerates cell proliferation, cell migration, and cell invasion via down-regulating G protein-coupled receptor kinase 6 (GRK6) expression. In addition, M2 macrophage exosomal miR-3917 also enhances tumor growth in vivo [58]. In lung tumor cells, hampering serine/threonine kinase 16 (STK16) reduces cell viability and stimulates cell apoptosis via blocking the AKT1 signal pathway. miR-181a-5p is enriched in M1 macrophages. In addition, miR-181a-5p binds to 3′UTR of ETS proto-oncogene 1 transcription factor (ETS1) [59]. The miR-181a-5p-enriched exosomes released by M1 macrophages can be transmitted to H1975 cells. Then, in H1975 cells, exosomal miR-181a-5p impedes cell viability and promotes cell apoptosis through impairing the ETS1-STK16-AKT1 axis [59]. Compared to M1 macrophage exosomes, miRNA let-7b-5p expression is decreased in M0 macrophage exosomes. Exosomal let-7b-5p from M1 macrophages suppresses cell proliferation and enhances cell apoptosis via repressing the G-protein gamma 5 subunit (GNG5) in A549 and H1299 cells [60]. As mentioned above, exosomal miRNAs from M2 macrophages always serve as oncogenic factors and promote lung cancer cell proliferation, cell migration, and cell invasion. On the contrary, exosomal miRNAs from M1 macrophages inhibit lung cancer cell biology.

#### 3.1.2. Exosomal miRNAs from Lung Tumor Cells Can Be Transferred into Macrophages

miR-19b-3p expression is higher in lung tumor cell-secreted exosomes than in lung tumor cells. miR-19b-3p targets protein tyrosine phosphatase receptor type D (PTPRD). Lung cancer cell-derived exosomal miR-19b-3p raises miR-19b-3p levels in macrophages. Then, miR-19b-3p up-regulation reduces PTPRD to promote M2 polarization of macrophages through the phosphorylation of signal transducer and activator of transcription 3 (STAT3). Chen et al. indicate that miR-19b-3p knockdown abrogates lung cancer metastasis via attenuating macrophage M2 polarization [61]. miR-181b is highly expressed in the serum of NSCLC patients. Through the activation of the Janus kinase 2 (JAK2)/STAT3 signaling pathway, exosomal miR-181b generated from A549 cells facilitates the M2 polarization of macrophages. Eventually, exosomal miR-181b potentiates lung cell proliferation, cell migration, and cell invasion [62]. miR-146a can be found in H1299 cell-released exosomes. Exosomal miR-146a from H1299 cells can be transmitted into macrophages and then elevates miR-146a expression in macrophages. Exosomal miR-146a suppresses TNF receptor-associated factor 6 (TRAF-6) and IL-1 receptor-associated kinase 1 (IRAK-1) to repress M1 macrophage polarization, which in turn enhances cell invasion and cell proliferation in H1299 cells [63]. Hypoxic TME amplifies aggressiveness and metastasis in NSCLC. The prognosis for NSCLC may be improved by targeting hypoxia [64]. Exosomes from hypoxic lung carcinoma cells contain a high concentration of miR-21. Interferon-regulatory factor 1 (IRF1) is a downstream target of miR-21. Under hypoxia, lung cancer cell exosomal miR-21 restricts IRF1 expression, which stimulates M2 macrophage polarization and ultimately contributes to lung cancer cell proliferation [65]. miR-101 is down-regulated in blood samples collected from lung cancer patients who had high levels of hypoxia-inducible factor 1α (HIF1α). miR-101 targets and inhibits cyclin-dependent kinase 8 (CDK8) [66]. The suppression of exosomal miR-101 from hypoxic A549 cells in the co-culture system raises CDK8 expression and then increases IL1A and IL6 in macrophages, resulting in macrophage inflammation. In addition, miR-101 over-expression abrogates lung cancer growth and inflammation in mice [66]. miR-770 can be packaged into exosomes secreted by A549 cells. miR-770 over-expression significantly decreases mitogen-activated protein kinase kinase kinase 1 (MAP3K1) expression in macrophages [67]. Exosomes derived by miR-770 agomir-treated A549 cells increase miR-770 expression in macrophages. miR-770 up-regulation limits macrophage M2 polarization through MAP3K1, impeding cell invasion and cell migration of A549 cells. Moreover, exosomes expressing up-regulated miR-770 also attenuate lung tumor growth in vivo [67]. Trivedi et al. suggest that dual-targeted hyaluronic acid-based nanoparticles can transfect SK-LU-1 cells with wild-type p53 (wt-p53) and miR-125b expressing plasmid DNA, thus increasing p53 and miR-125b levels in exosomes obtained from plasmid DNA-transfected SK-LU-1 cells. Exosomal wt-p53 and miR-125b reprogram M2 TAMs and lead to M2 repolarization towards the M1 state [68]. In summary, exosomal miRNAs from lung cancer cells can modify macrophage miRNA expression. In addition, disturbing the expression of lung cancer cell-derived exosomal miRNAs can reprogram macrophages. Exosomal miRNAs from lung cancer cells often promote M2 macrophage polarization, which in turn encourages lung cancer cell proliferation, cell migration, and cell invasion.

### 3.2. The Impacts of Exosomal lncRNAs on Macrophage-Linked Intercellular Communication

#### 3.2.1. Exosomal lncRNAs from Macrophages Can Be Transferred into Lung Tumor Cells

Unphosphorylated Yes-associated protein (YAP) has been shown to translocate into the cell nucleus where it binds to the TEA domain transcription factor (TEAD) family, resulting in gene transcription. Both YAP cytoplasmic retention and YAP degradation can be induced by phosphorylated large tumor suppressor kinase 1/2 (LATS1/2) [69]. As a member of heterogeneous nuclear ribonucleoproteins (hnRNPs), RBMX (RNA-binding motif gene, X chromosome) supports homologous recombination [70]. LINC00273 has been shown to facilitate lung cancer EMT via sponging miR-200a-3p and inducing zinc finger E-box binding homeobox 1 (ZEB1) expression [71]. Exosomal LINC00273 of M2 macrophages interacts with NEDD4 to accelerate LATS2 degradation, contributing to YAP dephosphorylation. Ultimately, the activated YAP signal boosts RBMX transcription through TEAD4, which enhances miR-19b-3p packaging into exosomes secreted by lung cancer cells [61]. Through STAT3 phosphorylation, lung tumor-derived exosomal miR-19b-3p elevates LINC00273 levels in M2 macrophage exosomes. M2 macrophage-released exosomal LINC00273 strengthens lung cancer metastasis [61]. LncRNA AGAP2 antisense RNA 1 (AGAP2-AS1) expression is up-regulated in lung cancer tissues while miR-296 expression is down-regulated. miR-296, which targets notch homolog protein 2 (Notch2), is repressed by AGAP2-AS1. Exosomal AGAP2-AS1 from M2 macrophages strengthens radiation resistance and cell cytotoxicity mediated by NK cells via suppressing miR-296 and elevating Notch2 in lung cancer [72]. These findings suggest that M2 macrophage-derived exosomal AGAP2-AS1 contributes to radiotherapy immunity of lung cancer via the miR-296-Notch2 axis. Furthermore, in lung cancer patients, high levels of AGAP2-AS1 expression can predict poor outcomes [72]. Taken together, exosomal lncRNAs from macrophages can change miRNA expression in lung tumor cell exosomes, which in turn alters lncRNA expression in macrophage exosomes. Moreover, macrophage exosomal lncRNAs also modify miRNA expression in lung tumor cells, which can ultimately influence therapeutic efficacy.

#### 3.2.2. Exosomal lncRNAs from Lung Tumor Cells Can Be Transferred into Macrophages

Exosomes obtained from A549 cells have large amounts of the lncRNA FGD5 antisense RNA 1 (FGD5-AS1). Exosomal FGD5-AS1 from A549 cells induces M2 macrophage polarization, leading to increased A549 cell migration and invasion [73]. In comparison to BEAS-2B cells, A549 cells exhibit higher levels of lncRNA prostate cancer-associated transcript 6 (PCAT6). miR-326 mimics inhibit Krüppel-like factor 1 (KLF1) expression. PCAT6 aims to target miR-326. Exosomal PCAT6 from A549 cells induces M2 polarization of macrophages, which contributes to EMT, invasion, and migration via the miR-326-KLF1 axis in A549 cells [74]. H1299-released exosomes containing LINC00313 have been shown to increase LINC00313 expression in macrophages. miR-135a-3p is sponged by LINC00313 to boost STAT6 expression. Through the miR-135a-3p-STAT6 axis, exosomal LINC00313 impedes M1 polarization while stimulating M2 polarization in macrophages. Exosomal LINC00313 produced by lung cancer cells also promotes M2 polarization of macrophages and lung tumor growth in vivo [75]. Advanced NSCLC is frequently treated with osimertinib, an irreversible third-generation tyrosine kinase inhibitor (TKI) of the epidermal growth factor receptor (EGFR) [76]. LncRNA SOX2 overlapping transcript (SOX2-OT) is concentrated in exosomes isolated from H1975 cells. In macrophages, miR-627-3p, as a downstream target of SOX2-OT, interacts with the 3′UTRs of Smad2, Smad3, and Smad4. Exosomal SOX2-OT secreted by H1975 cells promotes macrophage M2 polarization through the miR-627-3p-Smads axis, which makes H1975 cells resistant to osimertinib [77]. According to one study, exosomal SOX2-OT produced by NSCLC cells also contributes to lung cancer bone metastasis [78]. Additionally, serum SOX2-OT has been found as a biomarker for the diagnosis and prognosis of NSCLC [79]. As one tumor suppressor gene, phosphatase and tensin homolog (PTEN) blocks the phosphatidylinositol 3-kinase (PI3K)/AKT pathway [80]. In exosomes released by H1299 and SPC-A1 cells that are resistant to chemotherapy drug docetaxel (DTX), lncRNA short nucleolar RNA host gene 7 (SNHG7) is abundantly expressed. Exosomal SNHG7 recruits cullin 4A (CUL4A) to increase PTEN ubiquitination, which can activate the PI3K/AKT pathway [81]. Finally, exosomal SNHG7 cause M2 macrophage polarization, which in turn worsens DTX resistance of lung adenocarcinoma [81]. LINC00963 expression is higher in lung adenocarcinoma tissues than that in normal lung tissues. Lung cancer cell-derived exosomal LINC00963 potentiates lung cancer growth via stimulating M2 macrophage polarization in vivo [82]. In summary, lung cancer cell-derived exosomal lncRNAs can act as ceRNAs to repress miRNA inhibitory effects on targeted genes, eventually influencing macrophage polarization. In addition, lung cancer cell-derived exosomal lncRNAs can promote M2 polarization of macrophages to potentiate lung cancer growth and therapeutic resistance.

### 3.3. The Impacts of Exosomal circRNAs on Macrophage-Linked Intercellular Communication

Exosomal circSHKBP1 expression is up-regulated in serum from NSCLC patients. By sponging miR-1294, circSHKBP1 induces pyruvate kinase M2 isoform (PKM2) mRNA expression [83]. A549-derived exosomes expressing circSHKBP1 increase the expression of M2 polarization markers (e.g., CD206, IL-10, IL-4, and Arg1) while decreasing the levels of M1 polarization markers (i.e., CD86, IL-12, TNF-α, and INF-γ) [83]. Exosomal circSHKBP1 represses miR-1294 expression as well as enhances PKM2 expression and glucose uptake in macrophages [83]. These suggest that exosomal circSHKBP1 from A549 cells contributes to macrophage polarization from the M1 to M2 state via the miR-1294-PKM2 axis. Exosomal circSHKBP1 also accelerates lung cancer growth, metastasis, and M2 macrophage infiltration in the NSCLC mice model. Furthermore, serum exosomal circSHKBP1 might function as a biomarker for NSCLC diagnosis and prognosis [83]. Blood-derived exosomes from individuals with lung cancer contain a lot of circPVT1. Compared to normal tissues, lung cancer tissues exhibit reduced levels of miR-124-3 expression [84]. circPVT1 sponges miR-124-3p and inhibits its expression. The epigenetic regulator enhancer of zeste homolog 2 (EZH2) is a target of miR-124-3p [84]. Through the miR-124-3p-EZH2 axis, exosomal circPVT1 from A549 cells stimulates M2 macrophage polarization, which potentiates lung cancer cell proliferation, cell migration, and cell invasion. Additionally, high blood exosomal circPVT1 expression is linked to shorter survival time in lung cancer patients [84]. circFARSA is found in high concentrations in NSCLC tissues. circFARSA accelerates PTEN degradation and activates the PI3K/AKT pathway [85]. Lung tumor cell-derived exosomal circFARSA promotes M2 macrophage polarization through the PTEN/PI3K/AKT pathway, thus strengthening lung cancer cell invasion and migration [85]. Plasma circFARSA serves as a promising noninvasive biomarker for the diagnosis of NSCLC [86]. Following surgical resection of malignant lesions, patients with lung adenocarcinoma have decreased levels of serum exosomal circZNF451. E74-like factor 4 (ELF4) potentiates IRF transcription [87]. Exosomal circZNF451 supplied by lung tumor cells interacts with the E3 ligase TRIM56 before inducing fragile-X-related protein 1 (FXR1) ubiquitination to activate ELF4/IRF4 pathway in macrophages [87]. Thereby, exosomal circZNF451 stimulates M2 macrophage polarization to abolish the proliferation and immune functions of CD8^+^ T cells. Consequently, tumor exosomal circZNF451 is favorable for immune-suppressed TME and represses immunotherapy efficacy in lung cancer. Additionally, serum exosomal circZNF451 can be used as a biomarker to identify patients with lung adenocarcinoma who have a poor prognosis [87]. Compared to normal cells, the expression of circ-ADRM1 is up-regulated in lung cancer cells. Exosomal circ-ADRM1 from lung cancer cells (A549 and PC-9 cells) can enhance the M2 polarization of TAMs, which can promote lung cancer cell migration and invasion [88]. In short, exosomal circRNAs from lung cancer cells change macrophage polarized phenotypes via the miRNA-mRNA axis, which in turn supports lung cancer cell proliferation, cell migration, and cell invasion. Furthermore, circulating exosomal circRNAs function as potential noninvasive biomarkers for the diagnosis and prognosis of lung cancer patients.

**Figure 1:**
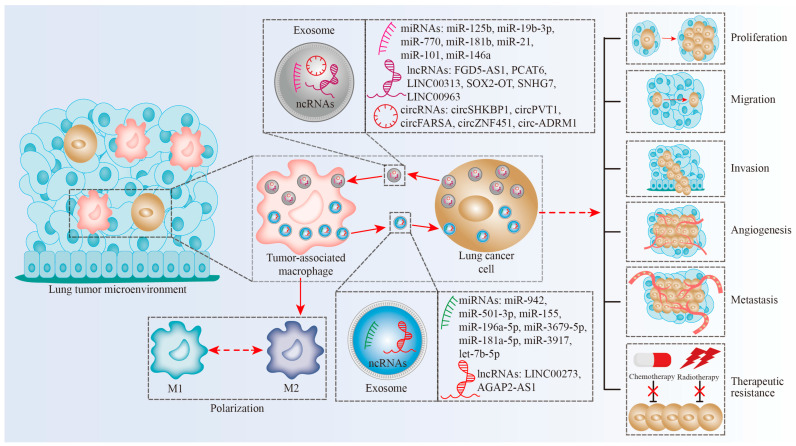
Exosomal ncRNAs modulate interplay between TAMs and lung cancer cells in lung TME. Tumor cells secrete exosomal ncRNAs to induce TAM polarization. TAMs deliver exosomal ncRNAs into lung cancer cells and influence cancer proliferation, migration, invasion, angiogenesis, metastasis, and therapeutic resistance.

Taken together, exosomal ncRNAs regulate intercellular communication between TAMs and lung tumor cells in lung TME (Figure 1), which can influence lung cancer proliferation, migration, invasion, angiogenesis, metastasis, and therapeutic resistance. Exosomal ncRNAs function as promoters or inhibitors in lung cancer (Table 1). Exosomal ncRNAs from lung tumor cells mainly induce M2 polarization of TAMs.

## 4. Exosomal ncRNAs Regulate Macrophage-Linked Intercellular Communication in Acute Lung Injury

ALI is a severe respiratory syndrome with the existence of hypoxemia. Acute respiratory distress syndrome (ARDS) is a serious form of ALI. ALI and ARDS are prevalent among critically ill patients [89]. In ALI, the resident lung cells can recruit inflammatory cells (i.e., neutrophils and macrophages) into the airway microenvironment and the inflammatory responses lead to lung injury [90]. There are many risk factors associated with ALI, including direct injury (e.g., pneumonia, gastric aspiration, pulmonary contusion, alveolar hemorrhage, etc.) and indirect injury (e.g., severe sepsis, transfusions, pancreatitis, and shock) [91]. Recently, LPS-induced in vitro and in vivo models have been widely used to study ALI pathogenesis [92]. M2 macrophages attenuate lung inflammation and inhibit ALI, whereas M1 macrophages promote lung inflammation and contribute to lung tissue injury [93]. LPS-treated macrophage-derived exosomes impede the cell viability of AECs [94]. Wang et al. indicate that exosomes from LPS-treated AMs facilitate inflammatory responses and lung tissue injury in ALI mice [95].

### 4.1. Exosomal ncRNAs from Immune Cells Can Be Transferred into Macrophages

Pyroptosis, an inflammatory cell death type mediated by caspase-1 or caspase-11, can elicit strong inflammatory responses to antagonize microbe infection. Excessive pyroptosis can result in a variety of inflammatory illnesses, including sepsis and autoimmune diseases [96,97]. The suppressor of cytokine signaling 1 (SOCS1) can reduce p65 stability to abrogate nuclear factor-kappaB (NF-κB) signaling [98]. Exosomes collected from TNF-α-treated polymorphonuclear neutrophils (PMNs) have higher miR-30d-5p expression than phosphate-buffered saline (PBS)-treated PMN exosomes. PMNs can deliver exosomal miR-30d-5p into macrophages and elevate miR-30d-5p expression in macrophages [99]. Subsequently, increased miR-30d-5p inhibits SOCS1 and SIRT1 to activate the NF-κB signal pathway in macrophages [99]. Exosomal miR-30d-5p eventually promotes M1 macrophage activation and macrophage pyroptosis, which can stimulate pulmonary inflammation and deteriorate sepsis-induced ALI. In septic mice, the injection of miR-30d-5p inhibitors can alleviate sepsis-induced pulmonary inflammation and lung injury [99]. In ALI monocyte-sourced exosomes, lncRNA colorectal liver metastasis-associated transcript 3 (CLMAT3) is diminished and C-terminal-binding protein 2 (CtBP2) is up-regulated. CtBP2 is a downstream target of CLMAT3 [100]. CtBP2 establishes a transcriptional complex with p300 and NF-κB, increasing the expression of pro-inflammatory cytokines, including IL-1β, IL-6, and TNF-α [100]. The exosomes loaded with CLMAT3 from ALI monocytes activate the CtBP2-p300-NF-κB complex in macrophages, resulting in promoting lung inflammation and contributing to ALI progression [100]. Exosomal ncRNAs from PMNs and monocytes can activate NF-κB signal in macrophages, thus promoting lung inflammation in ALI.

### 4.2. Exosomal ncRNAs from Stem Cells Can Be Transferred into Macrophages

Mesenchymal stem cells (MSCs) are defined as multipotent mesenchymal stromal cells with capacities to self-renew and differentiate into the mesodermal lineage (e.g., connective stromal cell, bone cell, fat cell, etc.) [101]. Bone marrow, umbilical cord blood, placenta, and adipose tissue are all sources of MSCs. Exosomes from MSCs dampen acute tissue inflammation and improve tissue regeneration [102]. Exosomes originating from adipose-derived stem cells (ADSCs) deliver mitochondrial ingredients into AMs to improve the mitochondrial function of AMs, which can lead to LPS-exposed AMs shifting from the M1 phenotype to the M2 phenotype, resulting in protecting mice against ALI [103]. Accumulative evidence suggests that macrophage autophagy (a lysosome-dependent degradation process) can repress or aggravate pulmonary inflammation and lung damage [104]. In exosomes from LPS-exposed bone marrow mesenchymal stem cells (BMSCs), miR-384-5p expression is raised. miR-384-5p targets and inhibits Beclin-1. BMSC exosomal miR-384-5p abolishes autophagy of AMs through Beclin-1 [105]. BMSC exosomes containing miR-384-5p attenuate pulmonary vascular permeability and pulmonary inflammation in LPS-treated mice, suggesting impeded lung injury [105]. Human umbilical cord mesenchymal stem cells (hUC-MSCs) produce a lot of exosomes that are rich in miR-451 [106]. miR-451 blocks PI3K/AKT signaling pathway via eliminating macrophage migration inhibitory factor (MIF) expression. Exosomal miR-451 from hUC-MSCs contributes to the M2 polarization of AMs via the MIF-PI3K-AKT axis, which hinders ALI development [106]. Exosomal ncRNAs from MSCs can reduce macrophage inflammation to inhibit ALI.

### 4.3. Exosomal ncRNAs from Epithelial Cells Can Be Transferred into Macrophages

miR-92a-3p is up-regulated in exosomes released by AECs after LPS stimulation. miR-92a-3p binds to PTEN 3′UTR [107]. Exosomal miR-92a-3p from LPS-treated AECs suppresses PTEN expression before activating the nuclear factor-kappaB (NF-κB) signal, which boosts pro-inflammatory factor (e.g., IL-1β, IL-6, and TNF-α) release in AMs, eventually resulting in lung inflammation and ALI [107]. For patients with severe respiratory failure or those who are under general anesthesia, mechanical ventilation is a crucial life-supporting treatment. Unsuitable mechanical ventilation, however, initiates or aggravates lung injury [108]. Exosomes from epithelial cells that have undergone cyclic stretching (CS) exhibit increased miR-21a-5p expression. CS-treated epithelial cell-derived exosomes transmit miR-21a-5p into recipient macrophages and elevate miR-21a-5p expression [109]. Exosomal miR-21a-5p subsequently reduces Notch2 and SOCS1 to enhance M2 macrophage polarization, which can exert protective anti-inflammatory effects in mechanical ventilation [109]. In response to mechanical ventilation in mice, over-expressed miR-21a-5p increases the percentage of M2 macrophages. These suggest a negative relationship between epithelial cell-derived exosomal miR-21a-5p and mechanical ventilation-related lung injury [109]. Exosomal ncRNAs from epithelial cells can show positive or negative effects on macrophage inflammation.

### 4.4. Exosomal ncRNAs from other Sources Can Be Transferred into Macrophages

miR-155 is highly expressed in serum exosomes extracted from ALI mice. Serum exosome-derived miR-155 from LPS-treated ALI mice stimulates macrophage proliferation via attenuating Src Homology-2 (SH2) domain-containing Inositol 5′-Phosphatase-1 (SHIP1) [110]. Serum exosomal miR-155 also potentiates macrophage inflammation via inducing NF-κB activation through targeting SOCS1 [110]. Serum exosomes loaded with miR-155 from ALI mice increase M1 macrophages, lessen M2 macrophages, and enhance lung inflammation in recipient mice. Additionally, inhibiting miR-155 can ameliorate septic ALI in vivo [110]. Exosomes from bronchoalveolar lavage fluid (BALF) of mice with LPS treatment show a reduction in miR-223-3p expression. BALF-derived exosomal miR-223-3p attenuates STK39 expression, thus strengthening autophagy and abrogating inflammation and apoptosis in macrophages challenged with LPS [111]. Moreover, in the ALI in vivo model, BALF exosomes carrying miR-223-3p mitigate pulmonary edema, cell apoptosis, and inflammatory factor production, suggesting minimized lung injury [111]. In Tie2^+^ AMs, the regulator of G protein signaling-1 (RGS1) depletion elevates IL10, SOCS3, and IL-4-stimulated Arg-1 and TGF-β1 expression. Endothelial cell-derived exosomal miR-223 down-regulates RGS1 to promote the anti-inflammatory and profibrotic effects of Tie2^+^ AMs [112]. In LPS-induced ALI mice, exosomes from endothelial cells raise the Tie2^+^ AM proportion, dampen lung injury, and improve blood oxygenation [112]. As mentioned above, exosomal ncRNAs from serum can display positive roles in macrophage inflammation during ALI. Exosomal ncRNAs from BALFs and endothelial cells can exhibit negative roles in macrophage inflammation in ALI.

## 5. Exosomal ncRNAs Regulate Macrophage-Linked Intercellular Communication in Pulmonary Fibrosis

Pulmonary fibrosis is a devastating lung disease featured by progressive and irreversible destruction of lung architecture, resulting in impaired gas exchange and respiratory failure [113]. Recently, mice with bleomycin (BLM) and silica treatment are often used to explore the disease progression of pulmonary fibrosis [114,115]. Current pathogenic theories proved that fibroblast to myofibroblast transition (FMT) plays a pivotal role in the pathogenesis of idiopathic pulmonary fibrosis, characterized by the abnormal generation of extracellular matrix (ECM) depositing in the lung parenchyma [116,117]. Silica-treated macrophage-derived exosomes promote fibroblast differentiation into activated myofibroblasts and augment fibroblast proliferation and migration through endoplasmic reticulum stress, which can lead to pulmonary fibrosis [118].

### 5.1. Exosomal ncRNAs from Macrophages Can Be Transferred into Fibroblasts

Sprouty homologue 1 (Spry1) is a negative regulator of the extracellular signal-regulated protein kinase (ERK)/mitogen-activated protein kinase (MAPK) signaling pathway [119]. miR-7219-3p in macrophage exosomes can be elevated by silica. After incubating with silica-treated macrophage exosomes, miR-7219-3p levels in fibroblasts are raised [120]. Macrophage exosomal miR-7219-3p accelerates FMT via attenuating Spry1 and activating the ERK/MAPK pathway. In addition, abrogating miR-7219-3p impairs silica-induced pulmonary fibrosis in mice [120]. miR-125a-5p is enriched in exosomes obtained from peripheral blood samples from patients with silicosis. Exosomal miR-125a-5p from silica-treated macrophages increases miR-125a-5p expression in fibroblasts [121]. Exosomal miR-125a-5p inhibits Smurf1 (an E3 ubiquitin-protein ligase) to activate the TGF-β signaling pathway, resulting in facilitating FMT [121]. The family with sequence similarity 13, member A (FAM13A) functions as a susceptibility gene associated with chronic obstructive pulmonary disease [122]. miR-328 expression is increased in macrophages of rats with pulmonary fibrosis. M2 macrophage exosomal miR-328 enhances the proliferation of pulmonary interstitial fibroblasts and raises fibrosis factor levels (collagen 1 A (COL1A), collagen 3 A (COL3A), and α-smooth muscle actin (α-SMA) in pulmonary interstitial fibroblasts via down-regulating FAM13A [123]. Moreover, silencing exosomal miR-328 of M2 macrophages dampens pulmonary fibrosis in vivo [123]. In pulmonary fibrosis rats, miR-129-5p is highly expressed. M2 macrophage exosomal miR-129-5p enhances fibroblast proliferation and stimulates pulmonary fibrosis via restraining STAT1 expression [124]. In exosomes obtained from the sputum and plasma of patients with idiopathic pulmonary fibrosis, miR-142-3p expression is elevated. In AECs and lung fibroblasts, over-expressed miR-142-3p represses cell proliferation and blocks the expression of ECM-related genes (e.g., *COL1A2*, *COL3A1*, and *TGF-β1*) via reducing TGF-β receptor 1 (TGFβ-R1) [125]. Macrophages deliver exosomal miR-142-3p into AECs and lung fibroblasts, which can inhibit profibrotic activation to impede pulmonary fibrosis [125]. It is indicated that up-regulation of PTEN-induced putative kinase 1 (PINK1) can restrain silica-induced lung inflammation and lung fibrosis in mice [126]. The expression of lncRNA MSTRG.91634.7 is decreased in silica-treated macrophages compared with normal macrophages. Macrophage-derived exosomal lncRNA MSTRG.91634.7 can increase PINK1 to suppress silica-induced fibroblast activation [126]. circRNA11:120406118|12040782 is highly expressed in serum exosomes isolated from silicosis patients. Inhibiting macrophage exosomal circRNA11:120406118|12040782 can repress fibroblast activation in response to silica treatment [127]. Exosomal ncRNAs from macrophages can be transferred into fibroblasts, which can influence fibroblast activation in lung fibrosis.

### 5.2. Exosomal ncRNAs from Other Sources Can Be Transferred into Macrophages

miR-27b-3p has been shown to induce synovial fibrosis in knee osteoarthritis [128]. Feng et al. report that repressing RGS1 promotes iNOS and IL-1β expression and elevates cathepsin and MMP activities in Flt3^+^ AMs [112]. Exosomal miR-27b-3p from type II AECs strengthens the pro-inflammatory and antifibrotic effects of Flt3^+^ AMs via decreasing RGS1. In addition, exosomes released by type II AECs increase the Flt3^+^ AM proportion and suppress BLM-exposed pulmonary fibrosis [112]. Recently, scientists can reprogram somatic cells to create pluripotent stem cells in a state similar to embryonic stem cells. These cells are called induced pluripotent stem cells (iPSCs) and can be used for regenerative medicine, disease modeling, and drug testing [129,130]. miR-302a-3p is up-regulated in iPSC exosomes compared to embryo fibroblast exosomes. Exosomal miR-302a-3p from iPSCs hinders M2 polarization of macrophages via targeting ten-eleven translocation 1 (TET1), resulting in repressing BLM-induced lung fibrosis [131]. Exosomal miRNAs from AECs and iPSCs can target macrophages to influence lung fibrosis.

## 6. Exosomal ncRNAs Regulate Macrophage-Linked Intercellular Communication in Asthma

Asthma is a chronic inflammatory respiratory illness affecting an estimated 334 million people in the world [132]. The symptoms of asthma include recurrent wheezing, chest tightness, cough, and shortness of breath. The main contributors to asthma pathogenesis include airway inflammation and airway remodeling [133]. Airway smooth muscle cells (ASMCs), the main structural component in the airway, affect airway inflammation via the secretion of various cytokines, chemokines, and growth factors [134]. M1 macrophages can release inflammatory factors to promote asthma. While M2 macrophages lead to inflammatory resolution and inhibit asthma [135]. Dong et al. suggest that exosomes from MSCs inhibit M1 polarization and promote M2 polarization of macrophages, thus lessening asthma in vivo [136]. miR-370 is down-regulated in lung tissues of asthmatic mice. Fibroblast growth factor 1 (FGF1) is a target of miR-370 [137]. M2 macrophage-derived exosomes containing miR-370 repress FGF1 to deactivate the MAPK/STAT1 signaling pathway, which can impede proliferation and inflammation in platelet-derived growth factor (PDGF-BB) treated ASMCs. In addition, M2 macrophage exosomal miR-370 also abrogates asthma progression in asthmatic mice [137]. Scorpion and centipede (SC) belong to insect Chinese medicine and can induce M2 macrophage polarization to relieve severe asthma [138]. M2 macrophage exosomes contain stronger miR-30b-5p expression than M1 macrophage exosomes. SC-induced M2 macrophage exosomes carrying miR-30b-5p reduce airway epithelial cell pyroptosis via restricting IRF7, resulting in restraining asthma progression [138]. EMT can be triggered by the TGF-β1/Smad3 signaling pathway and can support airway remodeling in asthma [139]. miR-21-5p is highly expressed in lung tissues of mice with ovalbumin (OVA)-induced asthma. miR-21-5p binds to Smad7 3′UTR. Exosomal miR-21-5p derived from LPS-exposed AMs activates the TGFβ1/Smad signaling pathway via down-regulating Smad7, which can lead to EMT in rat tracheal epithelial cells [140]. Shang et al. reveal that mmu_circ_0001359 expression is decreased in OVA-induced asthma mice. miR-183-5p can be sponged by mmu_circ_0001359 to augment FOXO1 expression [141]. Through the miR-183-5p-FOXO1 axis, exosomal mmu_circ_0001359 from ADSCs suppresses M1 macrophage polarization and stimulates M2 macrophage polarization, resulting in dampened airway remodeling in asthma mice [141]. In summary, exosomal ncRNAs from M2 macrophages or MSCs can inhibit airway remodeling to suppress asthma progression.

## 7. Discussion and Conclusions

Macrophages are one of the most prevalent immune cells found in lung tissues. Macrophages are highly plastic cells with different phenotypes and functions, which are influenced both by their origin and resident tissue microenvironment. M1 macrophages potentiate systemic inflammatory responses and restrain tumor progression. M2 macrophages restrict inflammation and accelerate the development of tumors. The specific components of exosomes determine whether they have supportive or inhibitory functions. ncRNAs are enriched in exosomes secreted by macrophages or other cell types. Cell-to-cell communication between macrophages and target cells is governed by exosomal ncRNAs. Exosomal ncRNAs modulate macrophage polarization as well as TME reprogramming. Exosomal ncRNAs regulate macrophage-linked intercellular communication to influence lung cancer and inflammatory lung diseases. Medical applications of ncRNAs in treating human illnesses have recently gained prominence. Some miRNA-based therapies are being examined in clinical trials, such as TargomiR (miR-16 mimic-based therapy) for malignant pleural mesothelioma [142] and Miravirsen (anti-miR-122 based therapy) for hepatitis C virus infection [143]. Accumulative evidence suggests that exosomes can be processed and used as drug carriers [144,145]. Immunotherapy is currently the most promising treatment strategy against cancers since tumor immune evasion is a crucial stage in the malignant growth of tumors and one of the main obstacles to it. Macrophage-targeting therapies are emerging immune-related treatments [146]. Presently, we discovered that Chinese medicine SC (scorpion and centipede)-induced M2 macrophages can transfer exosomal miR-30b-5p into airway epithelial cells and block pyroptosis, resulting in suppressing asthma [138]. This finding points to a potential lung dysfunction therapeutic approach employing macrophage exosomal miRNAs. These days, the rational design of therapies based on the exosomal ncRNA-macrophage axis has attracted widespread attention. We hope that this review will provide new ideas for more therapies such as SC (scorpion and centipede), thereby allowing us to treat lung disorders more effectively.

**Figure 2:**
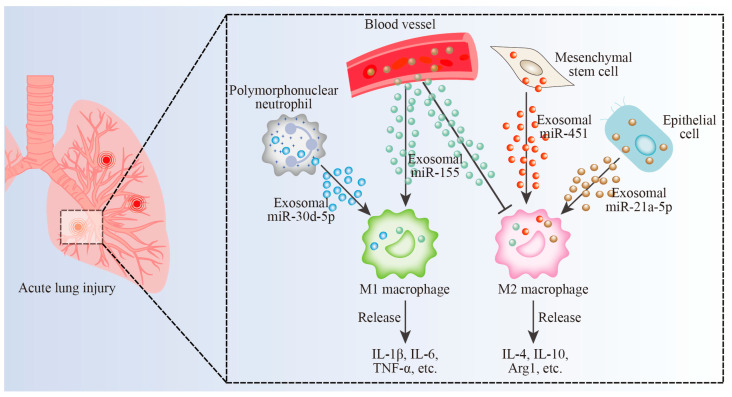
Exosomal miRNAs govern macrophage polarization in ALI. PMN-derived miR-30d-5p promotes M1 polarization. Blood-sourced miR-155 stimulates and represses M1 polarization and M2 polarization, respectively. MSC-derived miR-451 and epithelial cell-derived miR-21a-5p induce M2 polarization. M1 macrophages produce pro-inflammatory cytokines (i.e., IL-1β, IL-6, TNF-α, etc.) and M2 macrophages generate anti-inflammatory cytokines, including IL-4, IL-10, Arg1, etc.

In lung cancer, exosomal ncRNAs, such as miR-19b-3p [61], SNHG7 [81], and circFARSA [85], are transmitted into macrophages by tumor cells and alter macrophage polarization phenotypes. Polarized macrophages also deliver exosomal ncRNAs, including miR-942 [52] and AGAP2-AS1 [72], into cancer cells and support cancer migration, invasion, angiogenesis, metastasis, and therapeutic resistance. Exosomal ncRNAs from M2 macrophages always promote lung cancer progression. Exosomal ncRNAs (e.g., AGAP2-AS1 [72] and circZNF451 [87]) can be employed by lung tumor cells to facilitate immune evasion. Furthermore, exosomal ncRNAs can be served as lung cancer diagnostic or prognostic biomarkers, including circSHKBP1 [83], circPVT1 [84], and circZNF451 [87]. Exosomal ncRNAs also display positive or negative roles in inflammatory lung disorders (Table 2). In ALI, exosomal ncRNAs from MSCs [105,106], epithelial cells [107,109], PMNs [99], endothelial cells [112], monocytes [100], serum [110], and BALFs [111] can be transmitted into macrophages. Exosomal ncRNAs from different sources can control the M1/M2 polarization of macrophages (Figure 2) [99,106,109,110]. In pulmonary fibrosis, exosomal ncRNAs from macrophages can be transferred into fibroblasts [124,126,127]. In asthma, exosomal ncRNAs from macrophages can be transmitted into ASMCs [137,138] and epithelial cells [140]. MSCs possess the properties of tissue repair and tissue regeneration. Exosomal ncRNAs from MSCs always impede lung disorders, such as exosomal miR-384-5p from BMSCs [105] and miR-451 from hUC-MSCs [106] represses ALI, and exosomal mmu_circ_0001359 from ADSCs [141] suppresses asthma. Exosomal miR-302a-3p from iPSCs [131] reduces pulmonary fibrosis. M2 macrophage exosomal miRNAs inhibit asthma (miR-370 [137] and miR-30b-5p [138]) while promoting pulmonary fibrosis (miR-129-5p [124]). Furthermore, the ceRNA regulatory network, such as the PCAT6-miR-326-KLF1 axis [74], circSHKBP1-miR-1294-PKM2 axis [83], and mmu_circ_0001359-miR-183-5p-FOXO1 axis [141], also participates in lung disorders.

Agomirs up-regulate miRNA expression in exosomes and counteract the impacts of exosomes. For instance, miR-770 agomir administration elevates miR-770 expression in A549 exosomes and reverses the effects of A549 exosomes on TAMs [67]. Agomirs acquire the potential to reduce the formation of immunosuppressive TME and recover immune surveillance of macrophages. However, the applications of effective and clinically useful agomirs are difficult. Recently, inhalation-based siRNA administration has been considered as a potential lung injury treatment method [147]. The first siRNA therapeutic drug recognized for use in treating patients with hereditary transthyretin-mediated amyloidosis is called patisiran [148]. The clinical experience of the initial siRNA treatments provides fresh perspectives for further research into this therapeutic strategy for lung diseases. However, the clinical translation of ncRNA-based therapeutics has been challenged by treatment specificity, efficient delivery methods, and treatment tolerability [149]. Exosomes can regulate cell-to-cell communication through the delivery of proteins, lipids, and nucleic acids. Additionally, exosomes are considered natural carriers of ncRNAs and may act as an ideal delivery system [150,151]. However, the purification of exosomes and the stability of exosomes are paradoxical. Exosome drug delivery requires further exploration [152].

Presently, despite significant advancements in the study of lung cancer and inflammatory lung illnesses, the research based on the exosomal ncRNA-macrophage axis is still lacking. Exosomal ncRNAs are critical regulators of macrophage activation, polarization, inflammation, and recruitment. In lung cancer, studies are primarily conducted using NSCLC cell lines, and studies on SCLC cell lines and in lung cancer in vivo models are still limited. In inflammatory lung disorders, it is widely suggested that LPS-induced inflammation can mimic the injurious microenvironment. Animal models induced by LPS may be useful in investigating the pathological mechanism of lung injury.

## Figures and Tables

**Table 1:** Summary of effects of exosomal ncRNAs on lung cancer.

ncRNAs	Donors	Recipients	Targets/Pathways	Effects	Role	References
miR-942	Macrophages	SPC-A1, H1299	FOXO1-β-catenin axis	Increased migration, invasion, angiogenesis, and metastasis	Promoter	[52]
miR-501-3p	Macrophages	A549, SPC-A1	WDR82	Reduced apoptosis and elevated proliferation, migration, and invasion	Promoter	[53]
miR-155, miR-196a-5p	Macrophages	A549	RASSF4	Enhanced migration, invasion, EMT, and metastasis	Promoter	[55]
miR-3679-5p	Macrophages	A549	NEDD4L	Enhanced aerobic glycolysis and chemoresistance	Promoter	[56]
miR-3917	Macrophages	H1299, A549	GRK6	Enhanced proliferation, migration and invasion	Promoter	[58]
miR-181a-5p	Macrophages	H1975	ETS1-STK16-AKT1 axis	Reduced cell viability and enhanced apoptosis	Suppressor	[59]
let-7b-5p	Macrophage	A549, H1299	GNG5	Suppressed proliferation and enhanced apoptosis	Suppressor	[60]
miR-19b-3p	A549, H1975	Macrophages	PTPRD, STAT3	Enhanced metastasis	Promoter	[61]
miR-146a	H1299	Macrophages	TRAF-6, IRAK-1	Promoted invasion and proliferation	Promoter	[63]
miR-181b	A549	Macrophages	JAK2/STAT3 pathway	Enhanced proliferation, migration, and invasion	Promoter	[62]
miR-21	H1299	Macrophages	IRF1	Strengthened proliferation	Promoter	[65]
miR-101	A549	Macrophages	CDK8	Reduced macrophage inflammation and tumor growth	Suppressor	[66]
miR-770	A549	Macrophages	MAP3K1	Repressed invasion and migration	Suppressor	[67]
LINC00273	Macrophages	A549, H1975	NEDD4-LATS2-YAP axis	Increased metastasis	Promoter	[61]
AGAP2-AS1	Macrophages	A549, H157	miR-296-Notch2 axis	Induced radiotherapy immunity	Promoter	[72]
PCAT6	A549	Macrophages	miR-326-KLF1 axis	Enhanced EMT, invasion, and migration	Promoter	[74]
LINC00313	H1299	Macrophages	miR-135a-3p-STAT6 axis	Enhanced tumor growth	Promoter	[75]
SOX2-OT	H1975	Macrophages	miR-627-3p-Smads axis	Enhanced osimertinib resistance	Promoter	[77]
SNHG7	H1299, SPC-A1	Macrophages	PTEN/PI3K/AKT pathway	Increased DTX resistance	Promoter	[81]
circSHKBP1	A549	Macrophages	miR-1294-PKM2 axis	Strengthened tumor growth and metastasis	Promoter	[83]
circPVT1	A549	Macrophages	miR-124-3p-EZH2 axis	Enhanced proliferation, migration, and invasion	Promoter	[84]
circFARSA	A549	Macrophages	PTEN/PI3K/AKT pathway	Increased invasion and migration	Promoter	[85]
circZNF451	A549, H1299, H1395, H1975	Macrophages	TRIM56-FXR1-ELF4-IRF4 axis	Suppressed immunotherapy efficacy	Promoter	[87]

**Table 2:** Summary of effects of exosomal ncRNAs on inflammatory lung diseases.

Disease	ncRNA	Donors	Recipients	Targets/Pathways	Effects	Role	References
ALI	miR-30d-5p	PMNs	Raw264.7 macrophages	SOCS1, SIRT1, and NF-κB pathway	Enhanced lung inflammation	Promoter	[99]
	miR-384-5p	BMSCs	NR8383 macrophages	Beclin-1	Repressed lung injury	Suppressor	[105]
	miR-451	hUC-MSCs	NR8383 macrophages	MIF-PI3K-AKT axis,	Restrained lung injury	Suppressor	[106]
	miR-92a-3p	RLE-6TN cells	NR8383 macrophages	PTEN, NF-κB signal	Strengthened lung inflammation	Promoter	[107]
	miR-21a-5p	MLE-12 cells	Raw264.7 macrophages	Notch2, SOCS1	Suppressed lung injury	Suppressor	[109]
	miR-155	Serum	Raw264.7 macrophages	SHIP1, SOCS1, and NF-κB pathway	Increased lung inflammation	Promoter	[110]
	miR-223-3p	BALF	NR8383 macrophages	STK39	Attenuated lung injury	Suppressor	[111]
	miR-223	Endothelial cells	Tie2^+^ AMs	RGS1	Repressed lung injury	Suppressor	[112]
	CLMAT3	Monocytes	U937 macrophages	CtBP2-p300-NF-κB complex	Enhanced lung inflammation	Promoter	[100]
Pulmonary fibrosis	miR-7219-3p	RAW264.7 macrophages	NIH-3T3 cells	Spry1, ERK/MAPK pathway	Enhanced FMT	Promoter	[120]
	miR-125a-5p	RAW264.7 macrophages	NIH-3T3 and MRC-5 cells	Smurf1, TGF-β signaling pathway	Enhanced FMT	Promoter	[121]
	miR-328	Macrophages	Interstitial fibroblasts	FAM13A	Promoted lung fibrosis	Promoter	[123]
	miR-129-5p	Macrophages	Fibroblasts	STAT1	Enhanced lung fibrosis	Promoter	[124]
	miR-142-3p	Macrophages	A549 and MRC5 cells	TGFβ-R1	Attenuated lung fibrosis	Suppressor	[125]
	miR-27b-3p	Type II AECs	Flt3^+^ AMs	RGS1	Reduced lung fibrosis	Suppressor	[112]
	miR-302a-3p	iPSCs	RAW264.7 macrophages	TET1	Repressed lung fibrosis	Suppressor	[131]
	MSTRG.91634.7	Macrophages	MRC5 cells	PINK1	Reduced fibroblast activation	Suppressor	[126]
Asthma	miR-370	Macrophages	AMSCs	FGF1, MAPK/STAT1 signal pathway	Reduced asthma	Suppressor	[137]
	miR-30b-5p	RAW264.7 macrophages	Airway epithelial cells	IRF7	Restrained asthma	Suppressor	[138]
	miR-21-5p	NR8383 macrophages	Tracheal epithelial cells	Smad7, TGFβ1/Smad pathway	Enhanced EMT	Promoter	[140]
	mmu_circ_0001359	ADSCs	RAW264.7 macrophages	miR-183-5p-FOXO1 axis	Repressed airway remodeling	Suppressor	[141]

## Data Availability

Not applicable.

## Abbreviations

3′UTR: 3′untranslated region; ADSCs: adipose-derived stem cells; AECs: alveolar epithelial cells; AGAP2-AS1: AGAP2 antisense RNA 1; AKT: protein kinase B; ALI: acute lung injury; AMs: alveolar macrophages; ARDS: acute respiratory distress syndrome; Arg1: arginase-1; ASMCs: airway smooth muscle cells; BALF: bronchoalveolar lavage fluid; BLM: bleomycin; BMSCs: bone marrow mesenchymal stem cells; CDK8: cyclin-dependent kinase 8; ceRNAs: competitive endogenous RNAs; circRNAs: circular RNAs; CLMAT3: colorectal liver metastasis-associated transcript 3; CS: cyclic stretching; CtBP2: C-terminal-binding protein 2; CUL4A: cullin 4A; ECM: extracellular matrix; ELF4: E74-like factor 4; EMT: epithelial-mesenchymal transition; ERK: extracellular signal-regulated protein kinase; ETS1: ETS proto-oncogene 1 transcription facto; EVs: extracellular vesicles; EZH2: enhancer of zeste homolog 2; FAM13A: family with sequence similarity 13, member A; FGD5-AS1: FGD5 antisense RNA 1; FGF1: fibroblast growth factor 1; FMT: fibroblast to myofibroblast transition; FOXO1: forkhead box subfamily O 1; FXR1: fragile-X-related protein 1; GRK6: G protein-coupled receptor kinase 6; HIF1α: hypoxia-inducible factor 1α; hnRNPs: heterogeneous nuclear ribonucleoproteins; hUC-MSCs: human umbilical cord mesenchymal stem cells; IFN-γ: interferon-γ; IL-1R: IL-1 receptor; IL-4: interleukin-4; IMs: interstitial macrophages; iNOS: inducible nitric oxide synthase; iPSCs: induced pluripotent stem cells; IRF1: interferon-regulatory factor 1; KLF1: Krüppel-like factor 1; LATS1/2: large tumor suppressor kinase 1/2; lncRNAs: long noncoding RNAs; LPS: lipopolysaccharide; MAPK: mitogen-activated protein kinase; MHC II: major histocompatibility complex class II; MIF: macrophage migration inhibitory factor; miRNAs: microRNAs; MMP12: matrix metalloproteinase 12; mRNA: messenger RNA; MSCs: mesenchymal stem cells; MVs: microvesicles; ncRNAs: noncoding RNA; NEDD4L: neural precursor cell-expressed developmentally down-regulated gene 4-like; NF-κB: nuclear factor-kappaB; NK: natural killer; Notch2: notch homolog protein 2; NSCLC: non-small cell lung cancer; OVA: ovalbumin; PBS: phosphate-buffered saline; PCAT6: prostate cancer-associated transcript 6; PDGF-BB: platelet-derived growth factor; PI3K: phosphatidylinositol 3-kinase; PKM2: pyruvate kinase M2 isoform; PMNs: polymorphonuclear neutrophils; PTEN: phosphatase and tensin homolog; PTPRD: protein tyrosine phosphatase receptor type D; RASSF4: Ras association domain family member 4; RBMX: RNA-binding motif gene, X chromosome; RGS1: regulator of G protein signaling-1; SCLC: small cell lung cancer; SIRT1: sirtuin 1; SHIP1: Src Homology-2 (SH2) domain-containing Inositol 5′-Phosphatase-1; SNHG7: short nucleolar RNA host gene 7; SOCS1: suppressor of cytokine signaling 1; SOX2-OT: SOX2 overlapping transcript; Spry1: sprouty homologue 1; STAT6: signal transducer and activator of transcription 6; STK16: serine/threonine kinase 16; TAMs: tumor-associated macrophages; TEAD: TEA domain transcription factor; TET1: ten-eleven translocation 1; TGF-β: transforming growth factor-β; TGFβ-R1: TGF-β receptor 1; TKI: tyrosine kinase inhibitor; TME: tumor microenvironment; TNF-α: tumor necrosis factor-α; TUG1: taurine upregulated gene 1; WDR82: WD repeat domain 82; YAP: Yes-associated protein; ZEB1: zinc finger E-box binding homeobox 1.
